# The Impact of Leisure and Social Activities on Activities of Daily Living of Middle-Aged Adults: Evidence from a National Longitudinal Survey in Japan

**DOI:** 10.1371/journal.pone.0165106

**Published:** 2016-10-27

**Authors:** Takafumi Monma, Fumi Takeda, Haruko Noguchi, Hideto Takahashi, Nanako Tamiya

**Affiliations:** 1 Japan Society for the Promotion of Science, Tokyo, Japan; 2 Faculty of Health and Sport Sciences, University of Tsukuba, Ibaraki, Japan; 3 Faculty of Political Science and Economics, Waseda University, Tokyo, Japan; 4 Radiation Medical Science Center for the Fukushima Health Management Survey, Fukushima Medical University, Fukushima, Japan; 5 Faculty of Medicine, University of Tsukuba, Ibaraki, Japan; Taipei Veterans General Hospital, TAIWAN

## Abstract

This study investigated the effects of leisure and social activities on the ability of middle-aged adults to maintain activities of daily living (ADL), and whether performing these activities alone or with others contributed to the ability to perform ADL. The study used nationally representative longitudinal data of 22,770 adults in Japan, aged 50–59 years, who did not have limitations in performing ADL at the beginning of the 5-year survey period. The study considered six activity categories: two leisure activities (“hobbies or cultural activities” and “exercise or sports”) and four social activities (“community events,” “support for children,” “support for elderly individuals,” and “other social activities”). Multiple logistic regression analysis was used to examine the relation between participation in these categories at baseline and difficulties in ADL at the 5-year follow-up. The association between the extent of social interaction during these activities (“by oneself,” “with others,” or “both”) and difficulties in ADL was also investigated. The analysis yielded significant negative correlations between “exercise or sports” and difficulties in ADL for both men and women, and between “hobbies or cultural activities” and difficulties in ADL for women. However, these significant relationships occurred only when activities were conducted “with others.” The present findings might help prevent deterioration in middle-aged adults’ performance of ADL in Japan.

## Introduction

Although the Japanese have a relatively long lifespan, the gap between mean life expectancy and healthy life expectancy (i.e. the number of years that a person can expect to live in full health) is still 9.13 years for men and 12.68 years for women [1]. This gap refers to the period of experiencing limitations in daily activities and leads to individuals with a decreased quality of life who may become a burden to the national social security system. Therefore, extending healthy life expectancy and reducing the gap between mean life expectancy and healthy life expectancy is important for both public health and financial sustainability [1]. Maintaining health and avoiding unhealthy behaviors in midlife would reduce the risk of disabilities later in life [2]. Thus approaches that focus on healthy practices of middle-aged adults are necessary to extend healthy life expectancy.

Healthy life expectancy of Japanese individuals is calculated based on activity limitations in the Comprehensive Survey of Living Conditions (CSLC) [3], a nationally representative survey conducted by the Ministry of Health, Labour and Welfare (MHLW) [4]. Specific types of activity limitations were described including “activities of daily living (ADL),” “going out,” “work, household, and studies,” and “exercise” [3]. Among these, the ability to perform ADL is fundamental to maintaining an active daily life [5], which suggests that effective approaches to maintaining ADL should be widely encouraged.

A number of studies found that leisure activities (e.g. hobbies, cultural activities, exercise, and sports) and social activities (e.g. volunteering and community activities) help maintain ADL in older adults. For example, a 4-year longitudinal study in Japan reported that participation in hobby activities decreased the risk of functional disability among older adults [6]. Another Japanese study using 3-year longitudinal data reported that participation in hobby groups could effectively maintain older adults’ effectance, which represents higher-level functional capacity [7]. Furthermore, some meta-analyses have indicated that interventions involving exercise or sports can effectively maintain ADL in older adults [8, 9]. A longitudinal study targeting middle-aged adults also indicated a positive effect of exercise/sports on maintaining ADL [10]. Regarding social activities, longitudinal studies of older adults found that engaging in volunteer work was associated with decreased functional dependency [11, 12].

In addition, the presence of others participation in these activities may contribute to preventing deterioration in ADL performance (ADL deterioration). A study of older adults in Japan found that even when exercise was performed no more than once a week, the incidence of functional disabilities among those participating with others was lower than among those who exercised alone [13]. This finding suggests that social relationships may be a key factor in the prevention of functional disabilities. A systematic review reported that poor social relationships contributed to declines in functional status [14]. Thus, in addition to the positive effects of leisure and social activities themselves, social relationships developed through these activities are likely to promote maintenance of ADL.

However, no research has simultaneously examined the effects of middle-aged adults’ leisure and social activities on the maintenance of ADL; nor have studies investigated whether the effects of these activities are affected by the presence of other persons.

Our previous study investigated the effect of leisure and social activities on middle-aged adults’ mental health status, and considered the impact of the presence of other persons participating in the activity as an additional variable, using nationally representative longitudinal data from the Longitudinal Survey of Middle-aged and Elderly Persons (LSMEP) [15] conducted in Japan by the MHLW. It reported that leisure activities were related to mental health status at the 5-year follow-up, and that participation in leisure activities was strongly related to the presence of others [16]. While the previous study focused on the effect of leisure and social activities on mental health, this study examines the effect of these activities on ADL among middle-aged adults in Japan, using the LSMEP data.

## Methods

### Study population and procedure

This study used data extracted from the LSMEP, a nationwide, population-based survey, conducted in Japan by the MHLW each year since 2005. Respondents to the survey were extracted randomly through stratified two-stage sampling. First, 2,515 districts were selected at random from the complete set of 5,280 districts surveyed in the CSLC, conducted in 2004 by the MHLW. Subsequently, 40,877 residents of each selected district were randomly chosen from individuals aged 50–59 years, in proportion to the district’s total population.

In 2005, the first year of the survey, questionnaires were dropped off at the respondents’ homes by enumerators who returned to collect the self-reported surveys several days later. From the second year of the survey, the delivery method of the questionnaire changed from a “drop-off” to mail, and respondents returned the questionnaire by mail; the questionnaire was sent only to those who answered the questionnaire either last year, the year before, or both. The LSMEP did not recruit any new respondents since the survey’s first year.

We used data from the first and sixth surveys, in 2005 and 2010. Of the 40,877 people who received the original questionnaire, 34,240 responded to the survey in 2005 (response rate: 83.8%). Of these, we excluded 2,322 respondents with missing values regarding difficulties in ADL in 2005 and 2,737 respondents who had difficulties in ADL as of 2005 that could have prevented them from engaging in leisure and social activities, such as exercise or sports, leaving 29,181 eligible baseline respondents. Then, these respondents were contacted each year, provided they continued to complete the survey. As a result, the study sample comprised 22,770 respondents who completed the survey in 2010 (follow-up rate: 78.0%).

We obtained official permission to use the LSMEP from the MHLW based on Article 33 of the Statistics Act. We obtained the data in a fully anonymized and de-identified form. An ethical review was not required, based on the Japanese government’s Ethical Guidelines for Epidemiological Research [17].

### Measurements

#### Difficulties in ADL

Subjects’ difficulties in ADL were evaluated by responses to the question: “Do you have any difficulties in activities of daily living as follows: walking, getting up from a bed or a floor, sitting down and standing up from a chair, dressing, washing one’s hands or face, eating, toilet use, bathing, going up and down stairs, and carrying shopping bags?” Dichotomized responses (yes/no) were recorded.

#### Leisure and social activities

As with our previous study [16], two types of leisure activities were assessed: “hobbies or cultural activities (e.g. playing the Japanese game *go*, working with potted plants, traveling)” and “exercise or sports (e.g. walking, playing in a ball game),” and four types of social activities were assessed: “community events (e.g. neighborhood associations functions),” “support for children (e.g. assistance for children’s clubs),” “support for elderly individuals (e.g. support of housekeeping, transportation),” and “other social activities.” Respondents were asked whether they had participated in each activity during the year prior to the survey. For each question, those answering “yes” and “no” were categorized as active and inactive, respectively. Active respondents were asked to indicate the way in which they primarily participated in each activity: “by oneself,” “with families or friends,” “with co-workers (including former co-workers),” “in a neighborhood community association,” or “in a non-profit organization or public-interest corporation.” Respondents were permitted to select more than one response option. For the present study, respondents were categorized into three groups for each activity: “by oneself,” “with others” or “both” (i.e. doing the activity both “alone” and “with others”).

#### Demographic and socioeconomic status

Demographic and socioeconomic status included age (calculated from the month and year of birth), gender, living arrangement (i.e. living with a spouse, child(ren), father, mother, father-in-law, and mother-in-law, respectively), job status (employed or unemployed), personal income, and family care provision, which was evaluated by the question: “do you provide care for your family?” with dichotomized responses (yes/no).

#### Health status

Respondents’ answers regarding the presence of chronic diseases (diabetes, heart diseases, cerebral stroke, high blood pressure, hyperlipidemia, and cancer) were rated on a dichotomized scale (yes or no).

Mental health status was assessed using the Japanese version of the K6 scale [18], a screening tool for disorders involving psychological distress according to the Diagnostic and Statistical Manual of Mental Disorders (DSM-IV) [19]. Respondents answered six K6 items on a 5-point Likert scale, and responses for each item were transformed into scores ranging from 0–4 points. The total score ranged from 0 to 24. A higher total score corresponds to poorer mental health status. The respondents were divided into two groups, “good mental health status” (score below 5 points) or “poor mental health status” (5 points or above) according to the 5-point optimal cutoff for screening mood and anxiety disorders in Japan (with 100% sensitivity and 68.7% specificity) that has been used in previous Japanese studies [20, 21]. The Japanese version of the K6 has been validated [18], and the internal consistency reliability (Cronbach’s alpha) of the scale in this study was 0.87.

### Health behaviors

Health behaviors included smoking status (“smoker” or “non-smoker”) and alcohol drinking status (“drinker” or “nondrinker”).

### Statistical analysis

We used multiple imputation by chained equations to handle missing data in this study. Analysis of imputed datasets reduces the effects of potential biases introduced by missing data [22]. This method assumes that data are missing at random, and that any systematic differences between missing and observed values can be explained by differences in observed data [23]. Missing values were imputed according to a model comprising all other variables, and multiple imputation was used to create and analyze 10 multiple imputed datasets.

To investigate the relationship between the six types of leisure and social activities in the baseline survey and difficulties in ADL in the follow-up survey, two kinds of multiple logistic regression models were applied. Model 1 included the six types of activities (“hobbies or cultural activities,” “exercise or sports,” “community events,” “support for children,” “support for elderly individuals,” and “other social activities”) separately, as independent variables. Because the proportion of people who engaged in “support for children,” “support for elderly individuals,” and “other social activities” was low, the statistical power of these items was likely to be low. Therefore, the independent factors in Model 2 were two types of leisure activities (“hobbies or cultural activities” and “exercise or sports”) and a summarized index referred to as “social activities” indicated involvement in at least one of the four social activities. Furthermore, we conducted chi-square analyses and individual t-tests to compare the characteristics of active and inactive respondents with regard to leisure activities (participating in at least one of “hobbies or cultural activities” and “exercise or sports”).

Multiple logistic regression analyses were also performed to examine the impact of the sociality of activities (by oneself, with others, both ways, or not at all) on reducing difficulties in ADL. We used leisure and social activities that had positive effects in reducing difficulties in ADL in the prior logistic regression analyses as independent variables. Demographic and socioeconomic status (age, living arrangement, job status, personal income, and family care provision), health status (presence of chronic diseases and mental health status), and health behaviors (smoking status and alcohol drinking status) at baseline were controlled in the multiple logistic regression analyses. Logistic regression analyses, chi-square analyses, and individual t-tests were performed by gender because the proportion of respondents reporting participation in leisure and social activities in the baseline survey and limitations in ADL in the follow-up survey differed according to gender.

The level of significance for all analyses was set at *p* < 0.05. All statistical analyses were performed using IBM SPSS version 23.0.

## Results

A total of 17,856 respondents answered questions to all variables used in this study (78.4% of respondents in this study). The number of missing cases of each variable before multiple imputation by gender and by age (50–54 years old or 55–59 years old in the baseline survey) is shown in S1 and S2 Tables, respectively.

Descriptive statistics of the sample’s characteristics after multiple imputation are shown in Table 1. A larger proportion of women reported limitations in ADL in the follow-up survey compared to men. In addition, a larger proportion of women participated in “hobbies or cultural activities,” “support for children,” “support for elderly individuals,” and “other social activities” than did men; on the other hand, a greater percentage of men reported participation in “exercise or sports” and “community events” than did women.

**Table 1:** Characteristics of respondents after multiple imputation of missing values.

	Men (*n* = 11,029)				Women (*n* = 11,741)				P-value
	Mean (SE)		n (%)		Mean (SE)		n (%)		
**Demographic and socioeconomic status**									
Age	54.71	(0.03)			54.67	(0.03)			0.281^b^
Living arrangement:									
Spouse present			9619	(87.2)			9903	(84.3)	<0.001^c^
Child(ren) present			6970	(63.2)			7217	(61.5)	0.007^c^
Father present			1224	(11.1)			386	(3.3)	<0.001^c^
Mother present			2686	(24.4)			968	(8.2)	<0.001^c^
Father-in-law present			268	(2.4)			763	(6.5)	<0.001^c^
Mother-in-law present			615	(5.6)			1914	(16.3)	<0.001^c^
Employed			10510	(95.3)			8337	(71.0)	<0.001^c^
Personal income^a^	48.35	(0.65)			16.58	(0.32)			<0.001^b^
Family care provider			662	(6.0)			1204	(10.3)	<0.001^c^
**Health status**									
Diabetes			927	(8.4)			475	(4.0)	<0.001^c^
Heart diseases			342	(3.1)			163	(1.4)	<0.001^c^
Cerebral stroke			118	(1.1)			71	(0.6)	<0.001^c^
High blood pressure			2070	(18.8)			1764	(15.0)	<0.001^c^
Hyperlipidemia			1017	(9.2)			1018	(8.7)	0.145^c^
Cancer			119	(1.1)			199	(1.7)	<0.001^c^
Poor mental health			2529	(22.9)			2940	(25.0)	<0.001^c^
**Health behaviors**									
Smoker			5156	(46.7)			1301	(11.8)	<0.001^c^
Drinker			8241	(74.7)			3605	(32.7)	<0.001^c^
**Leisure and social activities**									
Hobbies or cultural activities			6593	(59.8)			7830	(66.7)	<0.001^c^
Exercise or sports			5460	(49.5)			5482	(46.7)	<0.001^c^
Community events			3655	(33.1)			3730	(31.8)	0.027^c^
Support for children			990	(9.0)			1218	(10.4)	<0.001^c^
Support for elderly individuals			921	(8.4)			1344	(11.4)	<0.001^c^
Other social activities			1448	(13.1)			1688	(14.4)	0.006^c^
**Difficulties in ADL at follow-up**			652	(5.9)			984	(8.4)	<0.001^c^

^a^ Ten thousand yen.

^b^ Independent *t*-test.

^c^ Chi-square test.

Table 2 presents the results of the multiple logistic regression analyses investigating the relationship between the six types of leisure and social activities at baseline and difficulties in ADL at follow-up. For men, “exercise or sports” decreased the risk of ADL difficulties at the 5-year follow-up (odds ratio [OR]: 0.70, 95% confidence interval [CI]: 0.58–0.84, *p* < 0.001) in Model 1. Model 2 also found a significant relationship between “exercise or sports” and fewer difficulties in men’s ADL (OR: 0.75, 95% CI: 0.62–0.89, *p* < 0.01).

**Table 2:** Results of multiple logistic regression analyses for the relationships between leisure and social activities and ADL.

		Model 1^a^			Model 2^a^		
		OR	95% CI	P-value	OR	95% CI	P-value
**Men**							
Hobbies or cultural activities	Active (ref. Inactive)	0.91	0.76–1.09	0.315	0.95	0.80–1.13	0.561
Exercise or sports	Active (ref. Inactive)	0.70	0.58–0.84	<0.001	0.75	0.62–0.89	0.001
Community events	Active (ref. Inactive)	1.02	0.83–1.24	0.875			
Support for children	Active (ref. Inactive)	1.33	0.88–2.01	0.172			
Support for elderly individuals	Active (ref. Inactive)	1.13	0.74–1.72	0.566			
Other social activities	Active (ref. Inactive)	1.19	0.84–1.69	0.315			
Social activities	Active (ref. Inactive)				1.11	0.93–1.33	0.246
**Women**							
Hobbies or cultural activities	Active (ref. Inactive)	0.80	0.69–0.94	0.005	0.84	0.72–0.98	0.023
Exercise or sports	Active (ref. Inactive)	0.79	0.68–0.92	0.002	0.86	0.75–1.00	0.050
Community events	Active (ref. Inactive)	0.98	0.82–1.16	0.807			
Support for children	Active (ref. Inactive)	1.61	1.21–2.13	<0.001			
Support for elderly individuals	Active (ref. Inactive)	1.32	1.01–1.74	0.045			
Other social activities	Active (ref. Inactive)	0.99	0.67–1.46	0.967			
Social activities	Active (ref. Inactive)				1.17	1.02–1.35	0.029

^a^ Adjusted for demographic and socioeconomic status (age, living arrangement, job status, personal income, and family care provision), health status (the presence of chronic diseases and mental health status), and health behaviors (smoking status and alcohol drinking status) at baseline.

Ref: reference; OR: Odds ratio; CI: Confidence interval.

With regard to women, “hobbies or cultural activities” (OR: 0.80, 95% CI: 0.69–0.94, *p* < 0.01) and “exercise or sports” (OR: 0.79, 95% CI: 0.68–0.92, *p* < 0.01) decreased the risk of difficulties in ADL at follow-up in Model 1. In contrast, “support for children” (OR: 1.61, 95% CI: 1.21–2.13, *p* < 0.001) and “support for elderly individuals” (OR: 1.32, 95% CI: 1.01–1.74, *p* < 0.05) increased this risk. Similarly, Model 2 showed that “hobbies or cultural activities” (OR: 0.84, 95% CI: 0.72–0.98, *p* < 0.05) and “exercise or sports” (OR: 0.86, 95% CI: 0.75–1.00, *p* < 0.05) decreased the risk of difficulties in ADL, whereas social activities (OR: 1.17, 95% CI: 1.02–1.35, *p* < 0.05) were associated with increased risk.

Subsequently, respondent characteristics were examined according to whether they engaged in leisure activities (Table 3). Significant differences in several variables were observed between active and inactive respondents with regard to leisure activities, because of the large sample size. In particular, the proportion of active respondents with poor mental health and smoking behavior was less than that of inactive respondents. Further, the average personal income of active men was much higher than that of inactive men. A smaller proportion of active women lived with their mother-in-law compared to inactive women.

**Table 3:** Characteristics of active and inactive respondents with regard to leisure activities.

	Men (*n* = 11029)						Women (*n* = 11741)					
	Leisure activities				χ^2^ or t	P-value	Leisure activities				χ^2^ or t	P-value
	Inactive		Active^a^				Inactive		Active^a^			
	n (%) or Mean (SE)		n (%) or Mean (SE)				n (%) or Mean (SE)		n (%) or Mean (SE)			
**Demographic and socioeconomic status**												
Age	54.64	(0.05)	54.76	(0.03)	1.583^c^	0.114	54.49	(0.05)	54.74	(0.03)	4.184^c^	<0.001
Living arrangement:												
Spouse present	2644	(85.4)	6975	(87.9)	12.717^d^	<0.001	2498	(83.6)	7405	(84.6)	1.557^d^	0.212
Child(ren) present	1974	(63.8)	4996	(63.0)	0.586^d^	0.444	1938	(64.9)	5279	(60.3)	19.460^d^	<0.001
Father present	343	(11.1)	880	(11.1)	0.000^d^	0.983	95	(3.2)	291	(3.3)	0.148^d^	0.701
Mother present	793	(25.6)	1893	(23.9)	3.755^d^	0.053	252	(8.4)	716	(8.2)	0.190^d^	0.663
Father-in-law present	89	(2.9)	179	(2.3)	3.604^d^	0.058	218	(7.3)	545	(6.2)	4.216^d^	0.040
Mother-in-law present	191	(6.2)	424	(5.3)	2.875^d^	0.090	575	(19.3)	1339	(15.3)	25.521^d^	<0.001
Employed	2930	(94.6)	7580	(95.6)	4.130^d^	0.042	2258	(75.6)	6079	(69.4)	40.507^d^	<0.001
Personal income^b^	41.55	(1.08)	51.00	(0.79)	6.594^c^	<0.001	15.70	(0.53)	16.87	(0.39)	1.613^c^	0.107
Family care provider	178	(5.8)	484	(6.1)	0.481^d^	0.488	276	(9.2)	928	(10.6)	4.511^d^	0.034
**Health status**												
Diabetes	239	(7.7)	688	(8.7)	2.627^d^	0.105	117	(3.9)	358	(4.1)	0.171^d^	0.679
Heart diseases	111	(3.6)	231	(2.9)	3.375^d^	0.066	45	(1.5)	118	(1.3)	0.409^d^	0.522
Cerebral stroke	39	(1.3)	79	(1.0)	1.465^d^	0.226	24	(0.8)	47	(0.5)	2.633^d^	0.105
High blood pressure	557	(18.0)	1513	(19.1)	1.682^d^	0.195	465	(15.6)	1299	(14.8)	0.926^d^	0.336
Hyperlipidemia	248	(8.0)	769	(9.7)	7.538^d^	0.006	205	(6.9)	813	(9.3)	16.527^d^	<0.001
Cancer	31	(1.0)	88	(1.1)	0.243^d^	0.622	50	(1.7)	149	(1.7)	0.011^d^	0.918
Poor mental health	819	(26.5)	1710	(21.6)	30.230^d^	<0.001	849	(28.4)	2091	(23.9)	24.296^d^	<0.001
**Health behaviors**												
Smoker	1692	(54.7)	3464	(43.7)	108.388^d^	<0.001	468	(15.7)	833	(9.5)	85.553^d^	<0.001
Drinker	2234	(72.2)	6007	(75.7)	14.698^d^	<0.001	775	(26.0)	2830	(32.3)	42.637^d^	<0.001

^a^ Respondents who participated at least one of "hobbies or cultural activities" and "exercise or sports."

^b^ Ten thousand yen.

^c^ Individual *t*-test.

^d^ Chi-square test.

Then, associations between social aspects of respondents’ engagement in “hobbies or cultural activities” or “exercise or sports” and difficulties in ADL were examined (Table 4). The “inactive” category was used as the reference. For men, “exercise or sports” was significantly related to reduced difficulty with ADL only when it was performed with others (OR: 0.68, 95% CI: 0.53–0.86, *p* < 0.01). Similarly, among women, both “hobbies or cultural activities” and “exercise or sports” were significantly and negatively correlated with difficulties in ADL only when it was performed with others (hobbies or cultural activities: OR: 0.80, 95% CI: 0.66–0.97, *p* < 0.05; exercise or sports: OR: 0.74, 95% CI: 0.57–0.95, *p* < 0.05).

**Table 4:** Multiple logistic regression analyses investigating the relationships between social aspects of activities and difficulties in ADL.

	Men				Women			
	n	OR^a^	95% CI	P-value	n	OR^a^	95% CI	P-value
**Hobbies or cultural activities (ref: Inactive)**								
By oneself					1560	0.78	0.60–1.02	0.069
With others					5884	0.80	0.66–0.97	0.022
Both					386	1.17	0.69–1.99	0.554
**Exercise or sports (ref: Inactive)**								
By oneself	1725	0.81	0.63–1.03	0.086	1788	0.90	0.73–1.09	0.281
With others	3387	0.68	0.53–0.86	0.002	3316	0.74	0.57–0.95	0.022
Both	348	0.79	0.23–2.74	0.705	377	1.15	0.48–2.74	0.752

^a^ Adjusted for demographic and socioeconomic status (age, living arrangement, job status, personal income, and family care provision), health status (the presence of chronic diseases and mental health status), and health behaviors (smoking status and alcohol drinking status) at baseline.

Ref: reference; OR: Odds ratio; CI: confidence interval.

## Discussion

In the previous Japanese study using LSMEP data, we showed that middle-aged women’s and men’s participation in leisure activities such as “hobbies or cultural activities” and “exercise or sports” with others was related to mental health status at the 5-year follow-up [16]. In this study, multiple logistic regression analyses of the selected LSMEP data revealed that “exercise or sports” for both genders and “hobbies or cultural activities” for women were related to ADL status at the 5-year follow-up only when these activities were conducted with others. This finding suggests that among leisure and social activities, participation in exercise or sports with others may effectively promote or maintain ADL and mental health in middle-aged men and women.

Our findings are consistent with several longitudinal studies that have reported that exercise or sports prevent ADL deterioration among older adults [24–26] and middle-aged adults [10]. On the other hand, some previous studies have suggested that hobbies or cultural activities decreased the risk of functional disabilities among older adults [6, 7]. The present study found significant longitudinal relationships between hobbies or cultural activities and reduced difficulties in ADL only among middle-aged women. This finding is of particular significance when designing intervention strategies for middle-aged women whose physical capacities do not permit them to engage in exercise or sports.

Furthermore, comparisons of active and inactive respondents, determined by whether they participated in leisure activities, yielded significant results. Active individuals were less likely to smoke and more likely to be in good mental health. In fact, poor mental health [27] and smoking [10] were considered to increase the risk of difficulties in ADL. Moreover, active men had significantly higher average personal incomes compared to inactive men. This is consistent with other studies that found a longitudinal impact of economic factors on ADL [28], with a greater effect on men’s health than on women’s health [29, 30]. Further, a significantly smaller proportion of active women—compared to inactive women—lived with their mother-in-law. Living with one’s mother-in-law may limit women’s available leisure time. These factors may contribute to preventing ADL difficulties by promoting participation in leisure activities.

Importantly, the present study indicated that participating in leisure activities with others might help maintain ADL. The findings suggest that only two baseline factors, participating in hobbies or cultural activities (among women) and exercise or sports (for both genders) with others were associated with reduced deterioration in ADL at follow-up. One possible explanation is that social relationships may enhance the benefit gained from these activities. One study showed that middle-aged individuals with consistently high levels of social relationships over a 34-year period leading up to old age had a lower risk of disability in their later years compared to those whose social relationships that decreased with time or remained at constant low to medium levels [31]. Another study on older adults in Japan showed that functional disability could be prevented more effectively when people were engaged in sports or exercise with others in organizations rather by exercising alone [13]. Our results suggest that social relationships provided by leisure activities, and not necessarily the activities themselves, might be key to maintaining ADL.

Nonetheless, these results may be influenced by differences in the sample size. The number of respondents participating in leisure activities with others was much larger than any other group. Although there were no significant relationships between engaging in leisure activities both “alone” and “with others” and difficulties in ADL, the sample size of this group was particularly small, implying that these results need to be considered carefully.

On the other hand, men’s and women’s participation in any of the four categories of social activities at baseline was not associated with limitations in ADL at follow-up. However, some studies targeting older adults have found such a relationship; for example, two longitudinal studies indicated that volunteering was associated with decreased functional dependency [11, 12]. This difference between results may be attributable to the considerably older sample in these studies. Older adults lose their social roles and their feelings regarding life being worth living with age [32, 33], and these in turn affect ADL [34] and other health outcomes [35]. Thus, engaging in social activities may provide opportunities to maintain social roles and one’s feeling that life is worth living, which may be especially important for ADL maintenance among older adults. However, in our study on middle-aged adults (50–59 years), 95.3% of men and 71.0% of women were employed at baseline. In other words, almost all respondents had other forms of regular social involvement. Therefore, it is understandable that participating in specific social activities does not contribute to preventing ADL deterioration in middle-aged adults.

Furthermore, women who engaged in social activities, especially those who support children and elderly individuals, may be more likely to have difficulties in ADL than those who did not engage in these activities. Previous studies have demonstrated a high prevalence of musculoskeletal pain—especially lower-back pain—in women with family-caregiving [36] or childcare [37, 38] responsibilities, and that this pain lead to difficulties in ADL [39]. In particular, women’s bone mass [40] and muscle mass [40] decreases rapidly with decreased estrogen levels post-menopause; further, postmenopausal women are at risk of osteoporosis and sarcopenia [41]. Thus, social activities may be risk factors for ADL deterioration via negative effects of musculoskeletal problems in middle-aged women.

The present study has several limitations related to the research process and sample. First, the most important limitation of this study is that it cannot claim a causal relationship between participation in leisure and social activities and maintenance of ADL. It is possible that causal relationships between these factors are the inverse of our hypothesis. Therefore, it is thus plausible that those who were more proficient in ADL at baseline were more likely to engage in various activities. Thus, directionality should be analyzed in future studies. Second, despite using multiple imputation to avoid the impact of missing variables, selection bias may exist. About 6,000 people did not respond to the baseline questionnaire, and about 6,000 respondents had dropped out at the end of 5 years. This limitation should be considered when generalizing the findings. Third, although we used multiple imputation methods to reduce the effect caused by attrition bias [42], it is impossible to prove that data are missing at random, rather than missing not at random. The examination of similarities of missing proportions between exposures and outcomes showed varied results. For example, in men, the missing proportion of exposures was 0–10.7% and that of outcomes was 3.1%; in women, this missing proportion of exposures was 0–7.9% and that of outcomes was 4.1%. Similarly, in respondents aged 50–54 in the baseline survey, the missing proportion of exposures was 0–9.3% and that of outcomes was 2.9%; in respondents aged 55–59 in the baseline survey, the missing proportion of exposures was 0–9.2% and that of outcomes was 4.2%. Because we did not have a complete set of the data, the influence of missing patterns on the results could not be determined. However, there is a possibility that missing values of some variables were not missing at random, and that the differences in missing patterns influenced the relationships between leisure and social activities and difficulties in ADL. Fourth, because participation in leisure and social activities was assessed using a dichotomized scale, the frequency or variety of each individual’s participation is unknown. Fifth, this study depended largely on respondents’ self-reported and retrospective answers regarding leisure and social activities, which may lead to less accurate results. Sixth, the LSMEP excluded hospitalized patients and residents of nursing homes who may have extraordinarily complex difficulties in performing ADL and do not to engage in leisure and social activities. The positive results in this study may therefore be underestimated. Finally, other confounders that are related to ADL, such as obesity or body mass index, [43] were not taken into account in this study.

Despite the above-mentioned limitations, the present study has several strengths. It used a large sample size and nationally representative data, and therefore reduced the effect caused by selection bias. Further, our study examined a wide range of leisure and social activities; it is more comprehensive than previous studies on middle-aged adults in Japan. Finally, in addition to our previous study, which recommends effective ways of promoting mental health, the present findings suggest that it is more effective for middle-aged adults to engage in leisure activities with others.

In conclusion, the present study found that exercise or sports for both genders and hobbies or cultural activities for women may have positive effects on ADL maintenance among middle-aged adults in Japan when these activities are performed with others. These findings might be useful when designing and developing strategies to encourage maintenance of ADL among middle-aged adults in Japan.

## Supporting Information

S1 Table The number of missing cases of each variable by gender. (DOCX)

S2 Table The number of missing cases of each variable by age. (DOCX)
