# Supplementary material for: The Impact of Leisure and Social Activities on Activities of Daily Living of Middle-Aged Adults: Evidence from a National Longitudinal Survey in Japan
Source: PLoS One. 2016 Oct 27;11(10):e0165106. doi: 10.1371/journal.pone.0165106 (PMC5082808; doi:10.1371/journal.pone.0165106)
Supplement: S1 Table — (DOCX) [file pone.0165106.s001.docx]

**S1 Table. The number of missing cases of each variable by gender.**

|  | Men (n=11029) | | Women (n=11741) | |  |
| --- | --- | --- | --- | --- | --- |
|  | n | (%) | n | (%) | P-value^a^ |
| **Demographic and socioeconomic status** |  |  |  |  |  |
| Gender | 0 | (0.0) | 0 | (0.0) | 1.000 |
| Age | 0 | (0.0) | 0 | (0.0) | 1.000 |
| Living arrangement: |  |  |  |  |  |
| Spouse | 18 | (0.2) | 30 | (0.3) | 0.149 |
| Child(ren) | 54 | (0.5) | 71 | (0.6) | 0.245 |
| Father | 54 | (0.5) | 71 | (0.6) | 0.245 |
| Mother | 54 | (0.5) | 71 | (0.6) | 0.245 |
| Father-in-law | 54 | (0.5) | 71 | (0.6) | 0.245 |
| Mother-in-law | 54 | (0.5) | 71 | (0.6) | 0.245 |
| Job status | 1 | (0.0) | 5 | (0.0) | 0.220 |
| Personal income | 1175 | (10.7) | 924 | (7.9) | <0.001 |
| Family care provision | 338 | (3.1) | 365 | (3.1) | 0.878 |
| **Health status** |  |  |  |  |  |
| Diabetes | 0 | (0.0) | 0 | (0.0) | 1.000 |
| Heart diseases | 0 | (0.0) | 0 | (0.0) | 1.000 |
| Cerebral stroke | 0 | (0.0) | 0 | (0.0) | 1.000 |
| High blood pressure | 0 | (0.0) | 0 | (0.0) | 1.000 |
| Hyperlipidemia | 0 | (0.0) | 0 | (0.0) | 1.000 |
| Cancer | 0 | (0.0) | 0 | (0.0) | 1.000 |
| Mental health | 517 | (4.7) | 540 | (4.6) | 0.753 |
| **Health behaviors** |  |  |  |  |  |
| Smoking status | 26 | (0.2) | 121 | (1.0) | <0.001 |
| Alcohol drinking status | 25 | (0.2) | 80 | (0.7) | <0.001 |
| **Leisure and social activities** |  |  |  |  |  |
| Hobbies or cultural activities | 628 | (5.7) | 706 | (6.0) | 0.310 |
| Exercise or sports | 628 | (5.7) | 706 | (6.0) | 0.310 |
| Community events | 628 | (5.7) | 706 | (6.0) | 0.310 |
| Support for children | 628 | (5.7) | 706 | (6.0) | 0.310 |
| Support for elderly individuals | 628 | (5.7) | 706 | (6.0) | 0.310 |
| Other social activities | 628 | (5.7) | 706 | (6.0) | 0.310 |
| **Difficulties in ADL at follow-up** | 342 | (3.1) | 477 | (4.1) | <0.001 |

^a^ Fisher's exact test
