# Supplementary material for: The Impact of Leisure and Social Activities on Activities of Daily Living of Middle-Aged Adults: Evidence from a National Longitudinal Survey in Japan
Source: PLoS One. 2016 Oct 27;11(10):e0165106. doi: 10.1371/journal.pone.0165106 (PMC5082808; doi:10.1371/journal.pone.0165106)
Supplement: S2 Table — (DOCX) [file pone.0165106.s002.docx]

**S2 Table. The number of missing cases of each variable by age.**

|  | Under 55 years old (n=10473) | | 55 years or above (n=12297) | |  |
| --- | --- | --- | --- | --- | --- |
|  | n | (%) | n | (%) | P-value^a^ |
| **Demographic and socioeconomic status** |  |  |  |  |  |
| Gender | 0 | (0.0) | 0 | (0.0) | 1.000 |
| Age | 0 | (0.0) | 0 | (0.0) | 1.000 |
| Living arrangement: |  |  |  |  |  |
| Spouse | 13 | (0.1) | 35 | (0.3) | 0.010 |
| Child(ren) | 52 | (0.5) | 73 | (0.6) | 0.368 |
| Father | 52 | (0.5) | 73 | (0.6) | 0.368 |
| Mother | 52 | (0.5) | 73 | (0.6) | 0.368 |
| Father-in-law | 52 | (0.5) | 73 | (0.6) | 0.368 |
| Mother-in-law | 52 | (0.5) | 73 | (0.6) | 0.368 |
| Job status | 1 | (0.0) | 5 | (0.0) | 0.228 |
| Personal income | 969 | (9.3) | 1130 | (9.2) | 0.872 |
| Family care provision | 303 | (2.9) | 400 | (3.3) | 0.124 |
| **Health status** |  |  |  |  |  |
| Diabetes | 0 | (0.0) | 0 | (0.0) | 1.000 |
| Heart diseases | 0 | (0.0) | 0 | (0.0) | 1.000 |
| Cerebral stroke | 0 | (0.0) | 0 | (0.0) | 1.000 |
| High blood pressure | 0 | (0.0) | 0 | (0.0) | 1.000 |
| Hyperlipidemia | 0 | (0.0) | 0 | (0.0) | 1.000 |
| Cancer | 0 | (0.0) | 0 | (0.0) | 1.000 |
| Mental health | 391 | (3.7) | 666 | (5.4) | <0.001 |
| **Health behaviors** |  |  |  |  |  |
| Smoking status | 70 | (0.7) | 77 | (0.6) | 0.741 |
| Alcohol drinking status | 39 | (0.4) | 66 | (0.5) | 0.078 |
| **Leisure and social activities** |  |  |  |  |  |
| Hobbies or cultural activities | 550 | (5.3) | 784 | (6.4) | <0.001 |
| Exercise or sports | 550 | (5.3) | 784 | (6.4) | <0.001 |
| Community events | 550 | (5.3) | 784 | (6.4) | <0.001 |
| Support for children | 550 | (5.3) | 784 | (6.4) | <0.001 |
| Support for elderly individuals | 550 | (5.3) | 784 | (6.4) | <0.001 |
| Other social activities | 550 | (5.3) | 784 | (6.4) | <0.001 |
| **Difficulties in ADL at follow-up** | 300 | (2.9) | 519 | (4.2) | <0.001 |

^a^ Fisher's exact test
